# Efficacy of an individual-tailored smoking cessation intervention APP among Chinese smokers: study protocol for a randomized controlled trial

**DOI:** 10.1186/s12889-023-16496-9

**Published:** 2024-01-02

**Authors:** Xiaoyun Xie, Lirong Liang, Yi Nan, Luge Zhang, Lin Xiao

**Affiliations:** 1 Tobacco Control Office, Chinese Center for Disease Control and Prevention, 155 Changbai Road Changping District, Beijing, 102206 China; 2 Department of Research on Tobacco Dependence Therapies, Beijing Institute of Respiratory Medicine and Beijing Chao-Yang Hospital, Capital Medical University, No.8 Gong-Ti-Nan-Lu, Chaoyang District, Beijing, 100020 China

**Keywords:** Mobile health, Smoking cessation, Randomized controlled trial

## Abstract

**Background and aims:**

Tobacco use has posed a tremendous public health problem for China. The Chinese government has taken great efforts to curb the tobacco epidemic. However, the existing smoking cessation services available in China are underused and have some limitations. Our research team intends to develop a smartphone smoking cessation application (SSC APP) and integrate it with the existing smoking cessation services. This study aims to assess the efficacy of the SSC APP developed by our research team through a randomized controlled trial (RCT).

**Methods:**

Current smokers who are motivated to quit within 1 month (n = 1000) will be recruited both online and offline, and all potential participants will register and complete the prescreening assessment online. Participants will be randomly assigned to either the intervention group (receiving the SSC APP and a self-help smoking cessation manual) or the control group (receiving a self-help smoking cessation manual only) using a block randomization method. This study will be a two-arm, single-blind, parallel-group RCT. Participants will be followed up after enrollment through online questionnaires or by phone call. The primary outcome is self-reported 6-month continuous abstinence. The main secondary outcomes include self-reported 7-day point-prevalence abstinence at each follow-up; self-reported 3-month continuous abstinence; reduction in the number of cigarettes smoked per day; and the number of recent quit attempts.

**Discussion:**

If this SSC APP proves to be effective, it could be integrated with the existing smoking cessation services and further facilitate smoking cessation at the population level in China.

**Trial registration:**

Chinese Clinical Trial Registry: ChiCTR2200062097, Registered July 22, 2022.

## Introduction

 Smoking is the single largest preventable cause of disease, disability, and death facing the world. In 2019, tobacco use accounted for 7.69 million deaths and 200 million disability-adjusted life-years loss worldwide [1]. China has approximately 308 million smokers [2], and 2.4 million people in China are dying of tobacco-induced diseases annually [1]. To curb the epidemic of tobacco in China, the Chinese government signed the World Health Organization Framework Convention on Tobacco Control (WHO FCTC), which suggests offering help to quit tobacco use. Besides, in 2019, the State Council issued the ‘Healthy China Action (2019–2030)’, in which the ‘Tobacco Control Action’ clearly stipulated the goal of “reducing the smoking rate of people over 15 years old to 20% by 2030” [3].

Numerous efforts have commenced for tobacco control purposes, one of which is the offering of professional smoking cessation aids. In China, smoking cessation services consist of brief smoking cessation interventions, smoking cessation clinics, and smoking cessation hotlines. Health professionals are suggested to perform brief smoking cessation interventions when smokers visit the hospital [4]. From 2010 to 2018, the proportion of smokers receiving advice from clinicians to quit smoking rose from 33.9 to 46.4% [5]. Besides, since 2014, the Chinese government has started to support the establishment of three Smoking cessation clinics (SCCs) in each province per year [6], as of 2021, China had more than 600 smoking cessation clinics providing professional cessation services for smokers [7]. SCCs generally are located within hospitals, and smokers could visit SCCs for intensive smoking cessation behavior intervention and drug treatment [8]. Smokers in China could also seek cessation services through smoking cessation hotlines, which provide theory-based telephone counseling and behavioral intervention. There are numerous regional and national quitlines offering voice consultation service and manual consultation for smokers in China.

However, these smoking cessation services are underused, with nearly 90% of smokers who have tried quitting over the past 12 months did not seek professional help [5]. Quitting smoking without professional help is hard to achieve as nicotine dependence is a stubborn addictive disease. Previous study indicates only 2-5% of untreated smokers can maintain long-term abstinence [9]. Fortunately, much can be done now. With the advent of internet technology, mobile health (mHealth) has been gradually deployed in the medical field. mHealth is a general term for the use of mobile phones and other wireless technology in medical care. The most common application of mHealth is the use of mobile devices to educate consumers about preventive healthcare services [10, 11]. Many research groups have dedicated themselves to exploiting this to facilitate smoking cessation [12–15], among which smartphone smoking cessation application (hereafter refers to ‘SSC APP’) is one hotspot as SSC APP has the potential to be an accessible, cost-effective, and efficient approach to facilitating smoking cessation and is in line with the development trend of time [16, 17].

Smartphones are rather popular in China, with 986 million people accessing the Internet through their mobile phones [18]. In addition, several studies suggest that Chinese smokers are open to SSC APPs and find it promising [19–22]. Compared with the existing smoking cessation services in China, SSC APP can be accessed at any time, is available anywhere through a smartphone, can reach smokers who want to quit smoking but do not want to seek professional support in person, and can increase the accessibility to evidence-based smoking cessation help. Additionally, we believe the existing smoking cessation services and SSC APP could complement and reinforce each other. Equipping automatic SSC APP with reliable professional health service providers could address smokers’ more specific questions while the deployment of SSC APP may diminish the workload of health professionals in clinics and on quit lines. Overall, there is an opening through which we could simultaneously help more people to quit smoking and optimize the existing smoking cessation services in China.

Due to cultural differences, SSC APPs proved effective in foreign countries may not perform well in China, hence the need to develop SSC APPs based on Chinese culture and relevant context. Several exploratory SSC APPs have been developed in recent years, including two WeChat Mini Programs (a lightweight application that can be accessed and used within the WeChat platform without requiring users to download or install any additional software a portable) developed by Chaoyang Hospital [23] and China-Japan Friendship Hospital [24] and one CBT-based SSC APP developed by Sir Run Run Shaw Hospital [25]. To further advance the development and utilization of SSC APPs, especially personalized ones, the Tobacco Control Office of the Chinese Center for Disease Control and Prevention, which oversees the existing smoking cessation services and is committed to controlling the epidemic of tobacco in China, plans to develop a theory-based, individual-tailored SSC APP and integrate it with the existing smoking cessation services such as smoking cessation clinics to better help smokers quitting smoking. To begin, a randomized controlled trial (RCT) will be conducted to evaluate the efficacy of this SSC APP. The gestation and development of the APP will be detailed in a future paper. This article intends to elaborate on the procedures of the RCT.

## Methods

### Design

This study will be a two-arm, single-blind, parallel-group RCT. It has been registered in the Chinese Clinical Trial Registry (trial registration number: ChiCTR2200062097).

### Study setting

This study will be completely conducted online with no restrictions on setting or location. A WeChat Official Account designed specifically for this study will be deployed to collect the baseline and follow-up data. The recruitment will be conducted from July 2023 to September 2023 or will be terminated when the target sample size is reached. The final 30-week follow-up period is projected to be completed by February 2024.

### Study population

To better understand the efficacy of this SSC APP, our study targets the largest potential user group. Thus, current Chinese smokers who are motivated to make a quit attempt within 1 month will be enrolled in this study.

### Inclusion criteria

- Aged 18 or above
- Current smoker, including daily smoker and occasional smoker
- Intend to quit smoking within 1 month
- Proficiency in smartphone
- Agree to take part in this study and is prepared to be assigned randomly to one of two treatment conditions
- Provide electronic informed consent

### Exclusion criteria

- Currently following other smoking cessation programs
- Has no access to an Android smartphone
- Inability to finish the study follow-up

### Sample size and power calculation

The proposed sample size is based on the primary outcome of self-reported 6-month continuous abstinence. Based on a meta-analysis conducted by Ybarra [26], the 6-month continuous abstinence for mobile phone-based smoking cessation intervention is 13.1%, compared with 6.4% in the control group. To detect a 2-sided significant difference between groups with a power of 80% and significance level of 5%, 309 participants in each arm are estimated to be required. Considering a 35.8% loss to follow-up in one study concerning SSC APP conducted in China [27], the final minimum sample size is 481 per group. Eventually, we set a target sample size at 1,000 people to cover the minimum sample size.

### Recruitment and informed consent

Recruitment will take place both online and offline. For online recruitment, Weibo, WeChat Official Accounts, web forums, and so on will be used to post the recruitment advertisements. With respect to offline recruitment, staff in local CDCs will assist in the distribution of recruitment posters and publicity. All recruitment channels will be attached with a Quick Response (QR) code of the WeChat Official Account of this research. After following the Official Account and completing the prescreening assessment, participants’ eligibility will be assessed automatically by computer algorithms. Baseline data, including sociodemographic characteristics and smoking history, will be collected in the prescreening assessment.

Eligible participants will receive a consent form with a summary of this study, including the study procedures, the risks and benefits, the intervention content of both the intervention group and control group, the potential of being allocated to either group, the compensation for participating this study and retaining in follow-up, how confidentiality is maintained, and explaining the data collection and data sharing procedures. Then participants will be asked to provide informed consent electronically by checking the box “I’ve read and understand the content of this study, and I agree to participate in this study.” To prevent unintended checks, a verification code will be required when participants decide to sign the informed consent. Besides, participants will be asked to provide a mobile phone number for short messaging system (SMS) reminders of research progress, follow-up questionnaires, as well as potential follow-up calls if they fail to complete the questionnaires on time.

### Randomization, allocation concealment and blinding

After enrollment, eligible participants will be assigned to either the control group or intervention group in a ratio of 1:1 (500 in each). Two stratification factors, each at two levels, will be used: age (≤ 40 years, > 40) and daily cigarette consumption (≤ 20 pack-years, > 20). A random permuted block sizes of 2, 4, or 6 will be used to achieve similar numbers of participants in both the intervention group and the control group.

Allocation concealment can be guaranteed as the random number sequence will be generated by a biostatistician who is not a member of the research team, and the random number sequence will be kept secret from researchers who are responsible for the recruitment.

As the interventions in two groups cannot be completely blinded in this study, this RCT will be single-blind in which all outcome assessors and data analysts will be blind to group allocation.

### Detailed description of intervention procedure and control group

This study aims to evaluate the efficacy of the individual-tailored smoking cessation intervention APP developed by the Tobacco Control Office. Thus, participants assigned to the intervention group will receive the APP and use it to guide their smoking cessation attempts, while participants in the control group will receive a self-help smoking cessation manual. To balance the effect of the self-help material, participants in the intervention group will receive them as well. In this way, this study could clearly determine the efficacy of this SSC APP.

#### Control group: self-help smoking cessation manual

“To help you stop smoking”, a 24 pages self-help smoking cessation manual developed by the Tobacco Control Office, is comprised of 12 sections, including the harms of smoking, the benefit of quitting smoking, the instruction on quitting smoking and medication usage, how to deal with setbacks, etc.

Participants in the control group will receive the self-help material alone to facilitate their smoking cessation attempts after randomization.

#### Intervention group: individual-tailored smoking cessation intervention APP group

This individual-tailored smoking cessation intervention APP was developed by the Tobacco Control Office. The Behavior Change Wheel (BCW) theory [28, 29] and Transtheoretical Model [30, 31] informed the function development of this APP as the Capability, Opportunity, and Motivation needed to quit smoking at each stage were analyzed thoroughly to justify the functions setting. This APP also embraces the key elements and concepts of cognitive behavioral therapy (CBT), including changing underlying beliefs about smoking and learning and practicing a variety of coping skills [32–34]. Also, during the development of this APP, suggestions and feedbacks from volunteer smokers were seriously considered. When the fundamental framework was formed, the Delphi method was exploited to solicit opinions and effective techniques from experienced smoking cessation experts and refine the APP iteratively. To recapitulate, this APP is theory-based and user-centered.

**Fig. 1:**
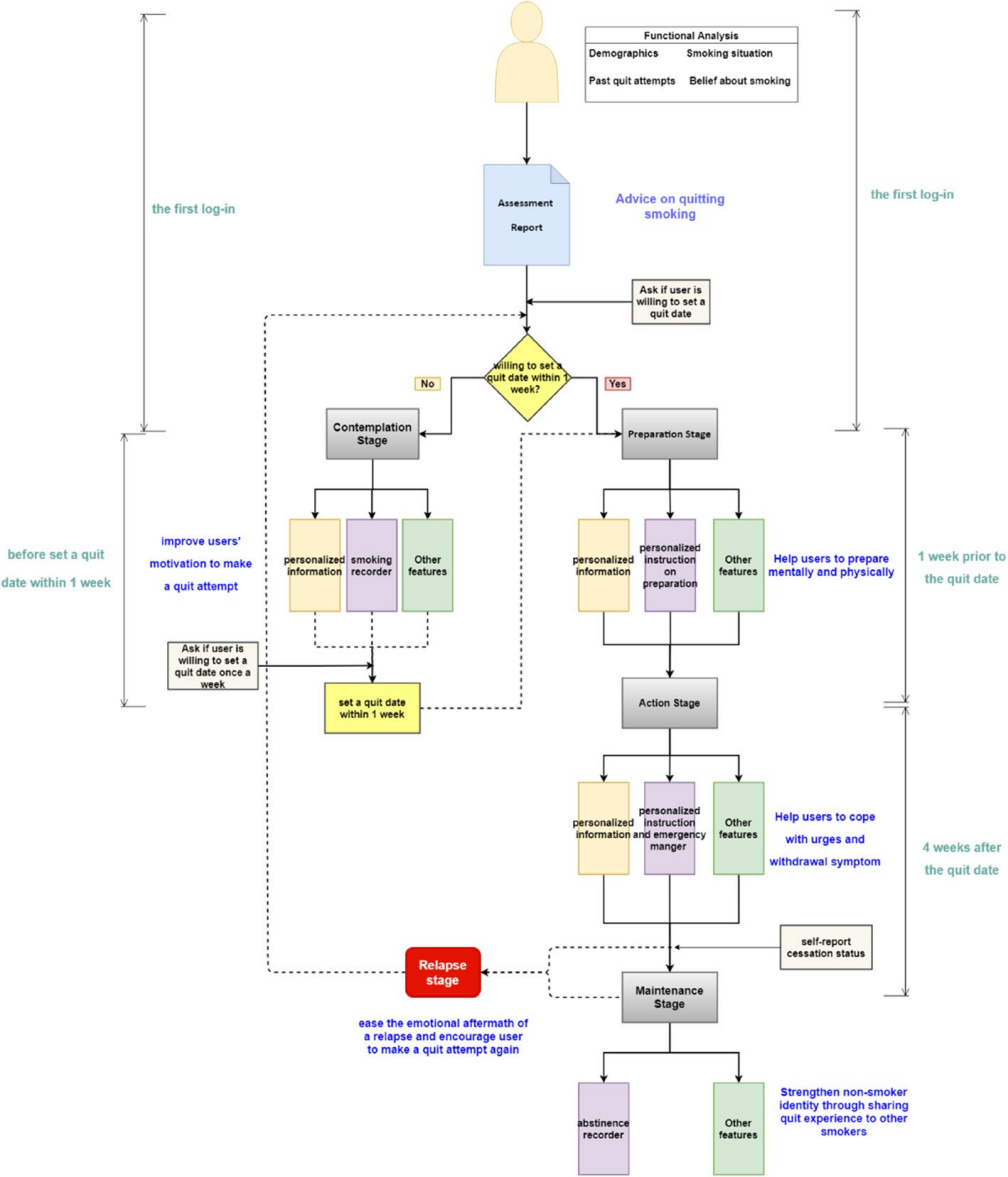
Functional framework of the SSC APP

At the first login, users will receive a thorough assessment questionnaire to evaluate their current stage, quit motivation, past quit attempts, current smoking usage, and conceptions about smoking. This functional analysis could set the stage for later individual-tailored interventions. Then, an assessment report will be generated accordingly, including users’ addiction level, current stage of behavior change, barriers to and facilitators of smoking cessation, personalized advice, and so on. Those in the preparation stage will be suggested to set a quit date and will receive stepwise guidance about the wherewithal to quit smoking. Considering the withdrawal symptoms and other challenges facing current smokers, users will receive tips and aids for coping with withdrawal symptoms, urges to smoke, and other problems during the first month. As for those in the contemplation stage, they may not yet be ready to make a quit attempt, hence they will be prompted to use this APP to record their smoking behavior, which provides an opening through which the APP could further intervene by sending specific motivational messages and presenting other smoking cessation-related material to them. These interventions may catalyze the transition from contemplation to preparation to quit. Along with these interventions and instructions, other functions will also be exploited to facilitate smoking cessation, such as an emergency help when smoking cravings occur. The gamification principle will be deployed to optimize the user experience as well. The functional framework of this SSC APP is shown in Fig. 1.

Participants in the intervention group will receive this APP and the self-help material to facilitate their smoking cessation attempts after randomization.

### Follow-up and data collection

Participants in both groups will be followed up for 6 months and receive electric follow-up questionnaires sent by the WeChat Official Account 1 month, 3 months, and 6 months after enrollment. SMS will be used to remind them to complete the questionnaires on time. Non-responders will receive up to 3 additional reminder messages or calls within 1 week following the initial invitation. Compensation will be paid to improve their willingness to respond and remain in this study. When the follow-up finishes, participants in both groups will be acknowledged for their participation and participants in the control group will be referred to our SSC APP if they have not quit smoking.

### Compensation

Participants in both groups will receive compensation through phone bill recharge after completing each follow-up questionnaire to stimulate their motivation to remain in the study. Considering participants are more likely to drop off at later follow-ups, the compensation will increase successively but the total financial value of the compensation will stand at 50 RMB (approximately US $7.86). Participants from both intervention and control groups will receive the same amount of compensation, and the answers to the questionnaires will not have any impact on the value of compensation received.

### Withdrawal from the program

It will be emphasized in the recruitment advertisement and the informed consent form that participation in this study is voluntary, and that participants will be free to withdraw at any stage of the study.

### Outcomes and measures

#### Primary outcome and measures

As we explained before, our SSC APP includes motivational protocols for those not ready to decide on a target quit date. In this study, we intend to explore the efficacy of the SSC APP as a whole, so the follow-up in this study will be tied to the date of intervention initiation. 6-month continued abstinence is selected as the primary outcomes. Considering there is no close contact or interaction between participants and the study team, biochemical verification may be infeasible as the SRNT guidelines have suggested [35], it will be self-reported. 6-month continued abstinence will be defined using the Russell 6 Standard [36] but without biochemical verification (i.e., tolerate fewer than five incidents of smoking from the quit date) and will be measured through the question “Have you smoked at all since (date of the start of the continuous abstinence period) ? A: No, not a puff; B: 1–5 cigarettes; C: More than 5 cigarettes.”

#### Secondary outcomes and measures

The following secondary outcome measures will be assessed in all participants at 1-month, 3-months, and 6-months after enrollment: 7-day point-prevalence abstinences, 3-month continuous abstinence, reduction in the number of cigarettes smoked per day, the number of quitting attempts, and attendance to other smoking cessation services.

The 7-day point-prevalence abstinence will be measured through the question “Have you ever smoked over the past 7 days?” The measurement of 3-month continuous abstinence is identical to that of 6-month continued abstinence as explained before. The number of cigarettes smoked per day and the number of quitting attempts will be asked at each follow-up. Attendance to other smoking cessation services will be measured through the question “Have you ever used any other professional smoking cessation services? If yes, please describe them briefly.”

As the participants in this study will be smokers willing to quit within 1 month, we presume this study will mainly reveal if our APP could help smokers in the preparation stage to quit successfully. However, since not every smoker proclaiming interest in quitting will make a quit attempt by their due date [37], this study could also explore whether our APP can increase the motivation of smokers to quit smoking. Therefore, we will specifically measure how many participants in each group have made attempts to quit 1 month after enrollment to explore the efficacy of our APP in increasing the motivation to make a serious quit attempt.

For those who relapse at the follow-up points, the reduction in the number of cigarettes smoked per day, the number of quit attempts, and attendance to other smoking cessation services will be considered as secondary outcomes and will be used to compare against the baseline data.

Furthermore, APP acceptability and engagement will be measured in the intervention group 1 month after enrollment by an adopted TAM (Technology Acceptance Model) questionnaire, which involves perceived usefulness and perceived ease of use [38]. Also, the frequency and duration of each login, the utilization of each specific function, and the compliance with guidance will be recorded automatically for further analysis. The full list of measurements is presented in Table 1.

**Table 1:**
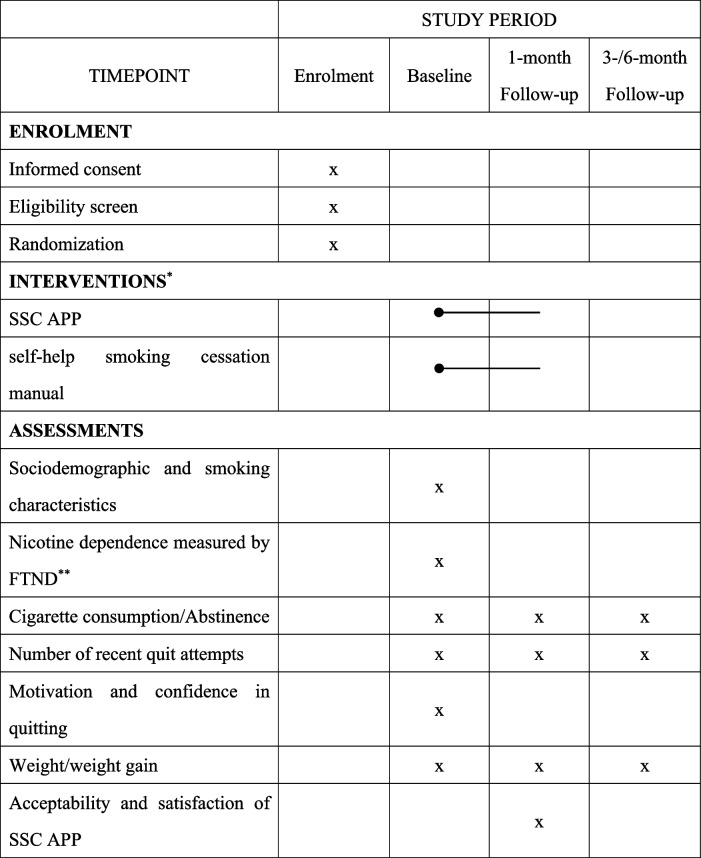
Schedule of enrollment and follow-up assessments

^a^ Schedule of enrollment and follow-up assessments

^b^ FTND, fagerström test of nicotine dependence

### Procedures

**Fig. 2:**
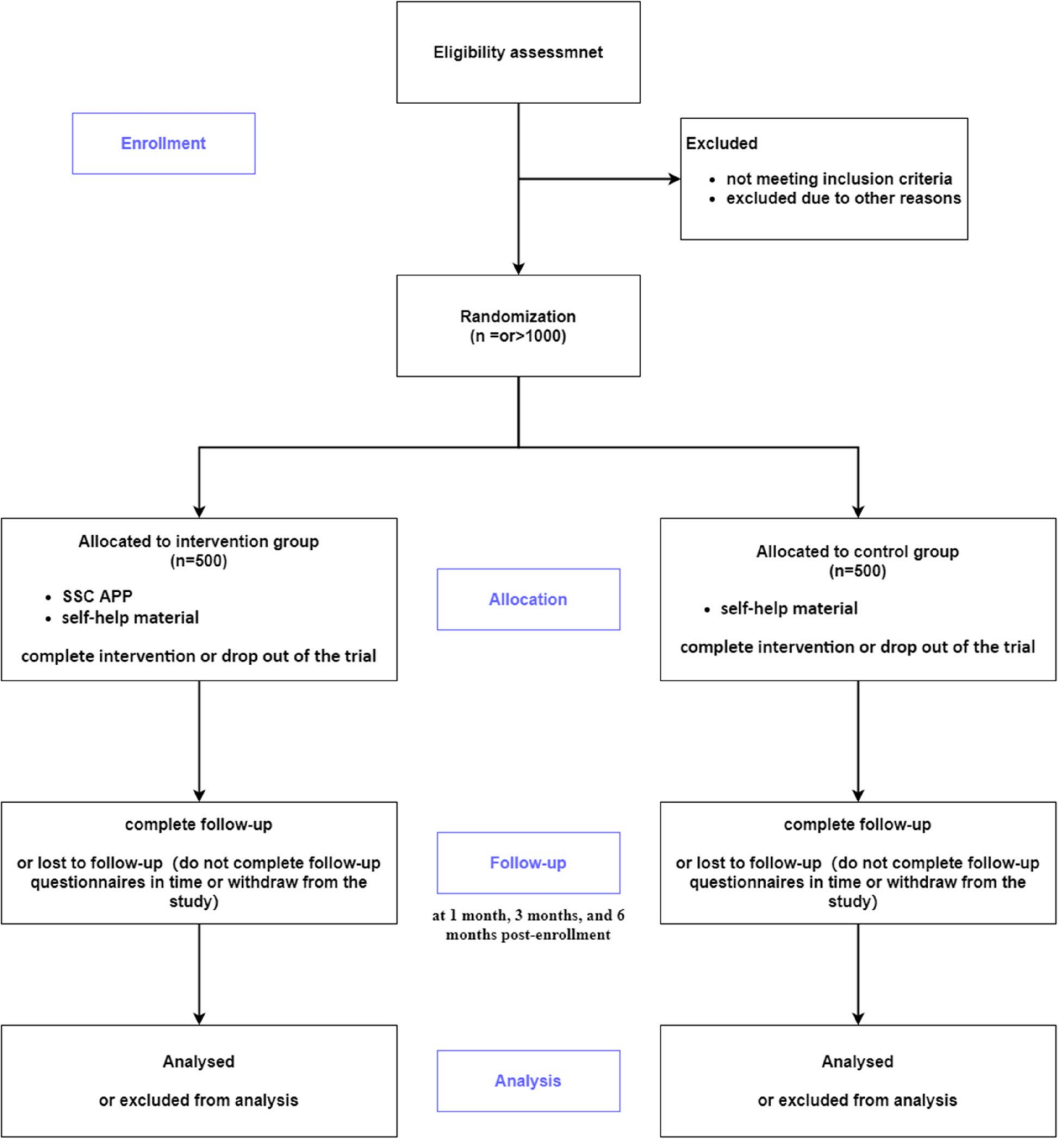
Trial flow diagram

Figure 2 shows the trial flow chart. Eligibility will be assessed at baseline and eligible participants will be allocated to either the intervention or control group in a ratio of 1:1 after stratification. Participants in both groups will receive self-help materials and the participants in the intervention group will receive the SSC APP in addition. Then participants in both groups will be followed up  for 6 months after enrollment.

### Statistical analysis

Upon completion of data collection, data will be cleaned and prepared for data analysis. Primary and secondary outcome variables will be checked for distribution, outliers, and missing patterns, and appropriate steps will be taken where necessary. All analysis will be conducted through SAS 9.4. Baseline demographics and smoking-related characteristics will be summarized and presented. Continuous variables (e.g. age) will be presented as numbers observed, means, and standard deviations. Categorical variables (e.g. marital status) will be presented as frequencies and percentages.

The main analysis will be focused on the comparison of smoking cessation rates in the intervention and control groups. This involves a comparison of the primary outcome variables and some secondary outcome variables including 7-day point-prevalence abstinence and self-reported continuous abstinence at each follow-up point. Independent t-tests and χ^2^ tests will be used to detect differences between the two groups depending on the type of variables. Logistic regression will be explored to assess group differences after adjusting for any baseline demographic or clinical characteristics that showed significant differences and variables that have been reported to be associated with smoking cessation [39].

Our analysis will be conducted based on intention-to-treat (ITT) principle and participants who are lost to follow-ups will be considered as continuing smokers with no reduction in cigarette consumption compared with baseline. The results from the analysis using the Multiple Imputation and Last Observation Carried Forward approaches to process missing data and per-protocol analysis will be conducted as sensitivity analyses. Subgroup analysis by baseline characteristics on quitting outcomes will be conducted by interaction terms.

### Ethics approvals and dissemination

Ethics approval (reference number: 202204) was obtained from the Ethical Review Committee of the Chinese Center for Disease Control and Prevention. Informed consent will be obtained from all the participants electronically. Results are expected to be published in peer-reviewed journals and presented at conferences.

### Safety

As most nicotine withdrawal symptoms are moderate and will subside within several weeks, we believe that this intervention is barely harmful to any participants. In case some participants experience any severe physical or mental withdrawal symptoms, professionals from the Chaoyang smoking cessation clinic will be available at any stage. Serious symptoms occurred will be recorded and reported.

### Confidentiality

The company responsible for software operations will sign a confidentiality agreement and complete the cyber security evaluation to avoid any data breach. All study-related data will be exported and stored securely on the research team’s encrypted computer under a coded identification number to maintain participant confidentiality. All data that contain personal identifiers, such as screening data for eligibility and informed consent forms, will be stored separately from study records identified by a code number. Only the principal investigator and research assistants will have access to the study data.

## Discussion

In recent years, SSC APPs have been booming worldwide. Hundreds of SSC APPs are circulating in the APP market on platforms like Google play and the Apple store, and they have attracted tremendous downloads [40, 41]. However, most APPs are not based on relevant scientific theories and there is little scientific evidence from randomized controlled trials on the impact of these APPs, especially in China, where no validated smoking cessation APP is publicly available now [40, 42]. Furthermore, due to the different cultural contexts, effective SSC APPs in foreign countries may not be well-worked in China [22].

This study will help us to evaluate the efficacy of an individual-tailored smoking cessation intervention APP developed by the Tobacco Control Office based on the BCW theory and the Transtheoretical Model. Ideally, it will help us understand how smartphones can be used to promote smoking cessation. If this APP proves to be effective, it could be integrated with the existing smoking cessation services and further facilitate smoking cessation at the population level. As a country with more than 300 million smokers, any amount of improvement could be amplified by the huge smoking population and yield considerable benefits.

As this study will be conducted online, several limitations lay ahead. First, it is unlikely to collect biochemical samples as participants are scattered remotely in various places. Thus, self-reported 6-month continued abstinence will be considered as an alternative to biochemically verified abstinence. Besides, without close contact or interaction, participants may be less likely to take this study seriously and thus more likely to be lost to follow-up. Hence, participants will be reminded through different approaches at the follow-up points and receive monetary incentives after completing follow-up questionnaires.

Overall, this study could shed some light on the efficacy of an individual-tailored smoking cessation intervention APP in China with the potential to address the existing and unmet demands of Chinese smokers. We predict this innovative study to be a promising attempt and may have major implications for smoking prevention policy and mobile health in general.

## Data Availability

Data generated and/or analyzed during the current study will be available from the corresponding author on reasonable request after the main results of the study have been published.
